# The abundance of bifidobacterium in relation to visceral obesity and serum uric acid

**DOI:** 10.1038/s41598-022-17417-3

**Published:** 2022-07-29

**Authors:** Hualan Gong, Hainv Gao, Qingye Ren, Jia He

**Affiliations:** Shulan (Hangzhou) Hospital Affiliated to Zhejiang, Shuren University Shulan International Medical College, Hangzhou, China

**Keywords:** Endocrinology, Health care

## Abstract

Gut microbiome has been shown to play a role in the development of obesity in recent studies. Most of these studies on obesity were based on the BMI classification criteria, which doesn't distinguish Visceral adipose tissue (VAT) from subcutaneous adipose tissue (SAT). Some studies showed that VAT has a higher risk of inducing metabolic diseases than SAT. This study focused on the visceral obesity defined by increased visceral fat area. The present study was designed to investigate the association of visceral obesity with gut predominant microbiota and metabolic status. This study included 372 healthy individuals from medical examination center in Shulan Hangzhou Hospital. Quantitative polymerase chain reaction (q-PCR) technique was used to detect ten kinds of gut predominant bacteria in fresh feces. Visceral fat area (VFA) was measured by the bioimpedance analyzer (INBODY720, Korea). The abundance of Bifidobacterium significantly decreased in the visceral obesity group. Compared with the lean group, Visceral obesity group had significantly higher levels of LDL, TG, FBG, serum uric acid (SUA) and lower levels of HDL. SUA was an independent impact factor for Bifidobacterium. SUA was negatively correlated with Bifidobacterium and positively correlated with VFA. In the mediation analysis, SUA showed significant mediation effect. SUA may be a mediating factor between decreased Bifidobacterium and increased VAT.

## Introduction

Obesity is a risk factor that seriously affects human health in the world. Obesity increases the risk of hypertension, diabetes, and cardiovascular disease^1^. BMI is the main defined criteria for obesity currently. It reflects the characteristics of body weight and total amount of adipose tissue, but it cannot reflect distribution characteristics of adipose tissue. Body adipose tissue is divided into subcutaneous adipose tissue (SAT) and Visceral adipose tissue (VAT). VAT refers to the fat surrounding the heart, liver, stomach and other organs. Several researches have confirmed that the relationship between obesity and cardiovascular diseases, metabolic diseases depended on the distribution of adipose tissue rather than its total amount^2,3^. VAT has been shown to have higher inflammatory activity than SAT^4^. Individuals with higher VAT have an increased prevalence of cardiometabolic disorders^5^ and insulin resistance^6^ compared to individuals with less VAT and relatively more SAT.

Gut microbiome is an important ecosystem in the human body. Most studies showed that there were significant gut microbiome disorder in obese individuals^7^. Compared with lean people, the abundance of Firmicutes was increased and the abundance of Bacteroidetes was decreased in the obesity people^8^. At the genus and species level, obesity individuals had higher count of Fusobacterium^9^, Enterococcus^10^, Prevotella^11^, and lower counts of Faecalibacterium^9^, Bifidobacterial than lean people^12,13^. Different Lactobacillus species are associated both with a lean and an obese status^14^.

At present, most studies on obesity and gut microbiota were based on BMI classification criteria. There were relatively few studies on visceral obesity and gut microbiota. Studying the association between visceral obesity and gut microbiota will help us remove the influence of SAT, which contributes to better study the relationship between obesity and metabolic state.

This study detected ten kinds of predominant gut microbiota, Investigate the association between these microbiota and visceral obesity, identify the meaningful gut bacteria, then study its relationship with metabolic markers: serum uric acid (SUA), triglycerides (TG), low-density lipoprotein (LDL), high-density lipoprotein (HDL) and fasting blood sugar (FBS). To explore the link between metabolic markers and gut microbiota, VAT.

## Materials and methods

### Study design

This study was carried out in the medical examination center department of Shulan Hangzhou Hospital from June 2018 to May 2021. All the participants were healthy, who underwent review of their medical history and physical examination. A complete anthropometrical assessment was carried out with measurement of height, weight, and body mass index (BMI). None had antibiotics and probiotics within 1 month before enrollment and acute disease or serious chronic disease in the past 3 months. Additional exclusion criteria were: (1) Secondary obesity: hypothalamus, pituitary disease, hypothyroidism, etc. (2) having severe heart, brain, liver and kidney disease, combined with tumors, immune or blood system diseases (3) Pregnancy or lactation women. The study was approved by the ethics committee of the Shulan (Hangzhou) Hospital and informed consent was obtained from all participants. All research was performed in accordance with relevant guidelines/regulations and the study was conducted in accordance with the Declaration of Helsinki.

We used q-PCR to detect the ten predominant gut microbes in their fresh stool, including probiotics: Lactobacillus, Bifidobacterium; Butyric acid producing bacteria: Faecalibacterium prausnitzii, Clostridium butyricum, Clostridium leptum, Eubacterium rectale; opportunistic pathogenic bacteria: Enterococcus, Enterobacteriaceae, Atopobium cluster; and Bacteroides. Body Composition Tester (INBODY720, Korea) was used to measure the visceral fat area (VFA). According to the VFA measurement results, all the individuals were divided into two groups: visceral obesity group (VFA ≥ 100cm^2^) and lean control group (VFA < 100 cm^2^)^15,16^. The fasting blood samples were extracted from antecubital vein using EDTA tubes, and sent to be immediately processed at the Laboratory of Shulan Hangzhou Hospital. The following indexes were detected using the Hitachi 7600 biochemical analyzer (Japan): TG, LDL, HDL, FBS and SUA.

### Use q-PCR to detect predominant gut microbiota

The information of PCR primers was shown in Supplementary Table S1 online. All oligonucleotide primers were synthesized by Gen Script (China). The ABI7500 real-time fluorescent PCR system (Applied Biosystems, USA) was used for the q-PCR amplification reaction. The amplification reaction contained 10uL of SYBRTM q-PCR master mix (Tong Chuang, China), 8 μL primers (0.2–0.6 μM), 2 μL template DNA, or 2 μL water (negative control), for a final volume of 20 μL. Each reaction was performed in triplicate, and the cycling threshold (ΔCt) < 0.5 between repetitions was required. Amplification was performed with the following temperature profiles: one cycle at pre-denaturation at 95 °C for 3 min, denaturation at 95 °C for 15 s, annealing and extension at 60 °C for 30 s, collection of fluorescence signals, a total of 40 cycles. The annealing and plate-reading temperatures for each primer pair are shown in Supplementary Table 1 online. The copy number of ribosomal DNA (rDNA) operons of targeted bacteria in crude DNA templates was determined by comparison with serially diluted plasmid DNA standards run on the same plate. Plasmid DNA standards were made from known concentrations of plasmid DNA that contained the respective amplicon for each set of primers. Bacterial count results were normalized to fecal bacteria count per gram (copies/g).

### Statistical analysis

SPSS software version 23.0 was used for statistical analysis. Normally distributed data were expressed as means and standard deviations. The t-test was used for comparison between groups, and the chi-square test was used for comparison of rates between groups. Non-normally distributed data were presented as median and interquartile range (IQR). Comparisons between groups were performed using the Mann–Whitney rank-sum test. Use Pearson correlation analysis to evaluate the correlation between gut microbiome and metabolic indicators. Multiple linear regression was used to analyze the independent correlation of gut microbiome with metabolic indicators after adjusting for confounding factors. For all tests, the statistical significance level was set at *p* < 0.05.

## Results

### Demographic and clinical metabolic characteristics

A total of 372 individuals were included, including104 subjects in the visceral obesity group (mean age 51.22 ± 10.60 years) and 268 subjects in the lean control group (mean age 49.39 ± 9.93 years). There were no significant differences in gender and age between two groups (P > 0.05). Visceral obesity group had significantly higher levels of BMI, SUA, TG, LDL, FBS and lower HDL (P < 0.05). (Table 1). Subjects were classified according to BMI as underweight (< 18. 5 kg/m^2^), normal weight (18.5–23.9 kg/m^2^), overweight (24.0–27.9 kg/m^2^), and obese (≥ 28.0 kg/m^2^) according to the Chinese standard^17^. There were 6 normal weight individuals in the visceral obesity group and 9 obese individuals in the lean control group.

**Table 1:** Personal and laboratory results in visceral obesity and lean group.

Variable	Visceral obesity (n = 104)	lean group (n = 268)	P值
Sex(male, proportion)	67 (64.4%)	167 (62.3%)	0.705
age (year)	51.22 ± 10.60	49.39 ± 9.93	0.119
BMI (kg/m^2^)	28.04 ± 3.18	23.03 ± 2.79	0.000*
Underweight(< 18. 5)	0	11	
normal weight (18.5–23.9)	6	152	
Overweight (24.0–27.9)	45	96	
Obese(≥ 28.0)	53	9	
FBS (mmol/L)	5.34 ± 0.95	4.93 ± 0.89	0.000*
HDL-C(mmol/L)	1.16 ± 0.31	1.27 ± 0.35	0.005*
LDL-C(mmol/L)	3.01 ± 0.86	2.85 ± 0.72	0.007*
TG (mmol/L)	2.39 ± 1.74	1.81 ± 1.36	0.003*
SUA (μmol/L)	364.43 ± 103.25	331.99 ± 89.79	0.003*

*FBS* fasting blood sugar, *HDL-C* high density lipoprotein, *LDL* Low-density lipoprotein, *TG* triglycerides, *SUA* serum uric acid.

*P < 0.05.

### Changes in the composition of the gut microbiome

**Figure 1:**
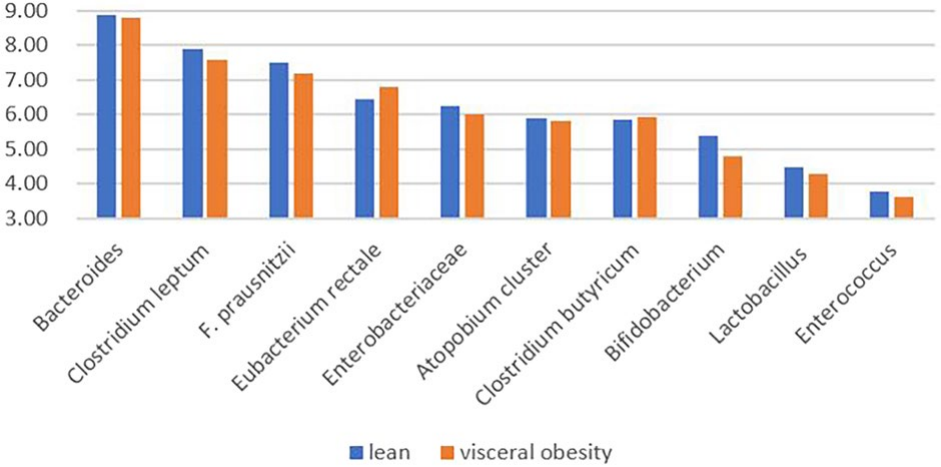
Median counts of ten bacteria in visceral obesity and lean groups. Common logarithm (lg) is used to convert bacterial counts. The median of Bifidobacterium in visceral obesity was 4.78. The median of Bifidobacterium in lean group was 5.36. The difference between two groups was statistically significant (P < 0.05).

**Figure 2:**
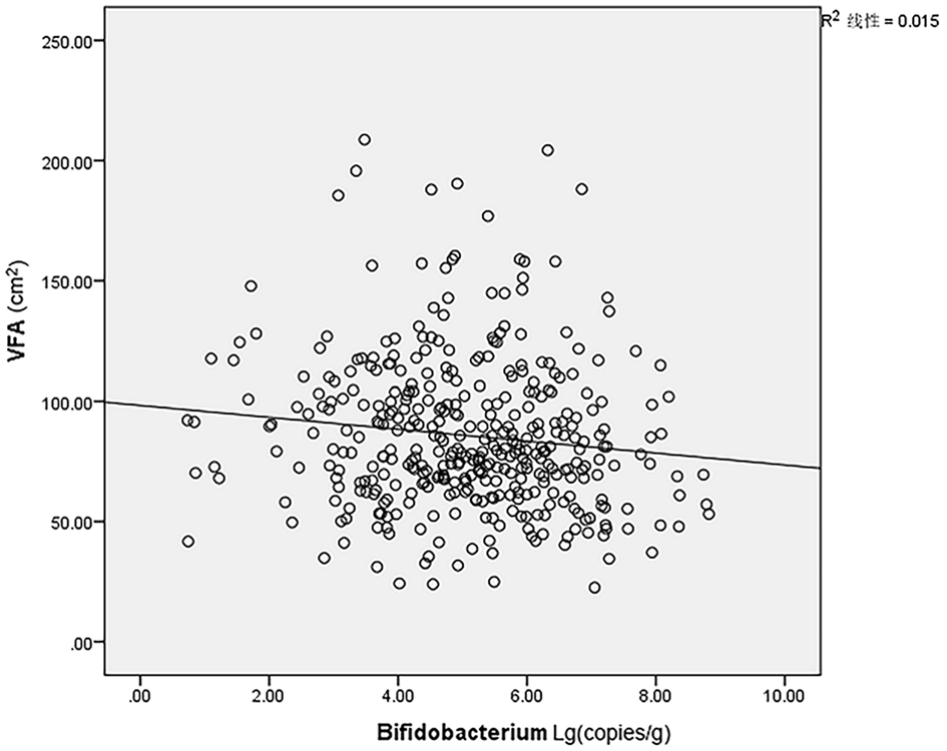
Correlation between Bifidobacterium and VFA.

Median count analysis showed that Bacteroides had the highest count of ten gut bacteria. Enterococcus had the lowest counts in both two groups. The counts of Eubacterium rectale and Clostridium butyricum in visceral obesity group was higher than lean group and the other eight bacteria in visceral obesity group were all lower than lean group (Fig. 1). The count of Bifidobacterium in the visceral obesity was significantly decreased, with a median of 6.08 × 10^4^ copies/g, and the median of Bifidobacterium in lean group was 2.30 × 10^5^ copies/g. The difference between the two groups was statistically significant (P < 0.05). Meanwhile, Bifidobacterium was negatively correlated with VFA (R = − 0.144, P < 0.01) (Fig. 2).

### Correlation analysis between Bifidobacterium, laboratory metabolic indicators and VFA

**Figure 3:**
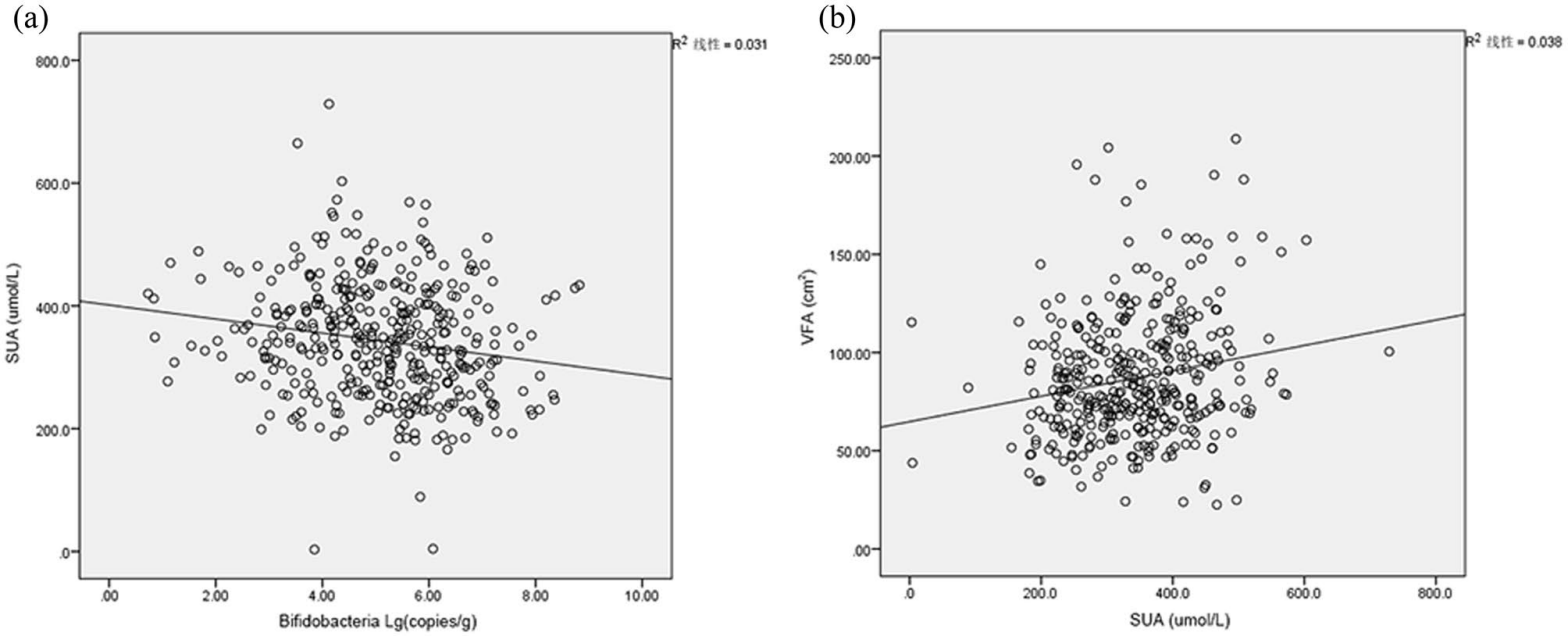
Bifidobacterium was negatively correlated with SUA (**a**) (R = − 0.176 P < 0.01). SUA was positively correlated with VFA (**b**) (R = 0.195, P = 0.000). *SUA* serum uric acid. *VFA* visceral fat area.

**Figure 4:**
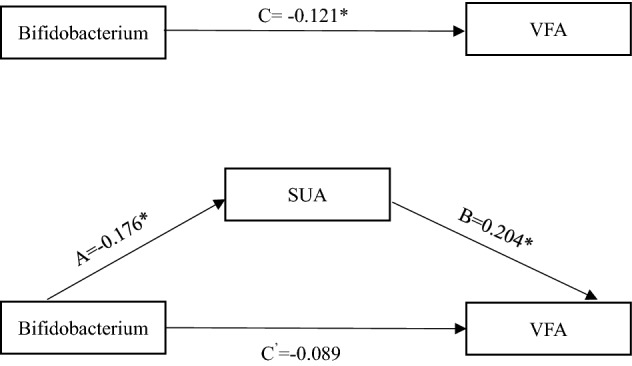
Proposed models that investigate mediated effects *P < 0.05.

Pearson correlation analysis was performed to analyze the correlation between Bifidobacterium and laboratory metabolic indicators: SUA, TG, LDL, HDL and FBS. Bifidobacterium was negatively correlated with SUA (R = − 0.176 P < 0.01) (Fig. 3) and positively correlated with HDL (R = 0.123, P < 0.05). Adjusting the age factor, the multiple linear regression showed that SUA was an independent impact factor for Bifidobacterial (β = − 0.151, P = 0.004) (Table 2). Pearson correlation analysis showed that SUA was positively correlated with VFA (R = 0.195, P = 0.000 (Fig. 3). The PROCESS Marco for SPSS was used to analyze the mediation effect of SUA. The mediation analysis was performed using one independent variable (Bifidobacterium), one dependent variable (VFA), and one mediator (SUA). When SUA was included in the mediation model, the standardized regression coefficient (β) for Bifidobacterium decreased from − 0.121 to − 0.089. It showed that SUA was the mediating factor between Bifidobacterium and VFA. Figure 4 illustrated the mediation model.

**Table 2:** Correlation and regression analysis of Bifidobacterium and metabolic indicators.

variables	Correlation analysis		Linear regression analysis	
	R	P	β	P
SUA(μmol/L)	− 0.176	0.000*	− 0.151	0.004*
FBS (mmol/L)	− 0.080	0.110	–	–
LDL (mmol/L)	− 0.049	0.322	–	–
TG (mmol/L)	− 0.064	0.199	–	–
HDL(mmol/L)	0.123	0.015*	0.095	0.074

*P < 0.05 Pearson correlation analysis showed Bifidobacterium was negatively correlated with SUA (R = − 0.176 P < 0.01). Bifidobacterium was positively correlated with HDL (R = 0.123, P < 0.05). Adjusting the age factor, multiple linear regression showed that SUA was an independent impact factor for Bifidobacterium (β = − 0.151, P = 0.004).

## Discussion

This study showed that individuals with visceral obesity had lower level of Bifidobacterium, and Bifidobacterium was negatively correlated with VFA SUA was an independent impact factor for Bifidobacterial. SUA was negatively correlated with Bifidobacterium and positively correlated with VFA. The mediation analysis showed that SUA may be a mediating factor between decreased Bifidobacterium and increased VAT.

Recent studies have showed that there was gut microbiota dysbiosis in obese individuals. Special gut microbiome leaded to fat deposits. Transplant gut microbiota from mice with diet induced obesity to lean germ-free mice, the germ-free mice developed more fat deposits^18^. The vast majority of gut microbiota belong to four main families (phyla): Firmicutes, Bacteroidetes, Proteobacteria and Actinobacteria^19^. At the genus and species level, obesity individuals had higher count of Fusobacterium, Enterococcus, Prevotella, and lower counts of Faecalibacterium, Bifidobacterial than lean people^9–13^. In this study, the counts of Faecalibacterium prausnitzii were also decreased in visceral obesity, but the difference was not significant. The counts of Bifidobacterium significantly decreased in visceral obesity, that was the same as the conclusion of previous studies about obesity based on BMI criteria^12,13^. In a recent study published in 2022 using whole-genome shotgun sequencing, Bifidobacterium longum showed a strong correlation with VFA. Visceral fat was more closely correlated with the gut microbiome compared with BMI^20^. In our study, according to the Chinese BMI standard. There were 6 normal weight individuals in the visceral obesity group and 9 obese individuals in the lean control group. The metabolic status of these individuals was interesting. It needs further study to understand its mechanism.

Gut microbiome has been shown to play a role in the development of obesity. Gut microbes contribute to the pathogenesis of obesity by fermenting indigestible dietary polysaccharides, producing short-chain fatty acids, and regulating energy homeostasis^21^. Supplementation of Bifidobacterium breve to high-fat diet-induced obese mice, significantly dose-dependently suppressed the accumulation of body weight and epididymal fat, and improved the serum levels of total cholesterol, fasting glucose and insulin^22^. Epididymal fat in the study was visceral fat. And this study did not detect serum uric acid. In another study, Supplementation of probiotic yogurt with Bifidobacterium lactis Bb12 decreased the level of serum uric acid^23^. Our study found that SUA was an independent impact factor for Bifidobacterium. SUA was negatively correlated with Bifidobacterium and positively correlated with VFA. The mediation analysis showed that SUA may be a mediating factor between decreased Bifidobacterium and increased visceral adipose tissue.

SUA is a product of purine nucleotide metabolism, mainly derived from exogenous diet and endogenous nucleic acids. A certain level of serum uric acid is considered to be a beneficial antioxidant, but excess uric acid is associated with various diseases, such as hypertension^24^, diabetes^25^, cardiovascular disease^26^. Multiple epidemiological studies have shown a positive correlation between visceral fat and serum uric acid levels^27,28^. High levels of serum uric acid can increase insulin secretion, thereby promote fat synthesis^29^. Uric acid can also directly promote fat synthesis in hepatocytes via ER stress-induced activation of SREBP-1c^30^. This suggests that controlling serum uric acid levels may reduce the accumulation of visceral fat.

Besides exogenous diet, the gut microbiota also plays an important role in SUA level. About one-third of uric acid is excreted through the gut^31^. The gut microbiota has gradually become a new target to study the pathogenesis of hyperuricemia. Transplant fecal microbiota of diet induced hyperuricemia rats into recipient rats, SUA levels were significantly increased in recipient rats^32^. The abundance of gut Faecalibacterium prausnitzii, Clostridium butyrate-producing bacterium, Bifidobacterium decreased in the gout people^33^. As a member of probiotics, Lactobacillus can reduce SUA levels by synthesizing uric acid degrading enzymes^34^. The mechanism of the association between Bifidobacteria and SUA was unclear. Several studies have found that Bifidobacterium can reduce endotoxin levels, reduce intestinal mucosal permeability, and have a protective effect on the intestinal mucosal barrier^35,36^. Normal intestinal mucosal barrier helps to prevent the translocation of intestinal bacteria or bacterial lipopolysaccharide (LPS) into the blood. Elevated LPS levels in the blood increased the risk of hyperuricemia^37^.

Currently the main and widely used medications for lowering serum uric acid are xanthine oxidase inhibitors such as allopurinol. Some people cannot tolerate these medications because of its side effects. Bifidobacterium is a kind of probiotic that has been widely used. Its clinical safety has been proven, almost no side effects. In addition to direct supplementation with Bifidobacterium, supplementation with specific prebiotics, such as chicory, could also help to reduce serum uric acid levels^38^. By lowering SUA levels, we can lower the accumulation of visceral adipose tissue, further reduce the risk of metabolic diseases caused by visceral obesity.

This study also has several limitations. First, we only detected ten gut bacteria. There is an interaction between the vast gut microbiota. It's easy to miss other meaningful gut microbiota. We need to further use 16S rRNA gene amplicon sequencing to detect gut microbiota in people with visceral obesity. Assess the species composition of the gut microbiota and its relative abundance information in visceral obesity. Then use q-PCR to analyze the role of specific gut bacteria. Second, this study was cross-sectional data, which cannot determine any cause-effect relationships. The interactions between SUA and the gut microbiome are complex and dynamic. Therefore, more longitudinal study data are needed to help understand the mechanism of the link between SUA and Bifidobacterium, VFA.

## Conclusions

This is a new perspective to study obesity and gut microbiota. Studying visceral obesity independently is beneficial to precisely prevent obesity-induced metabolic disease risk. The counts of Bifidobacterium significantly decreased in visceral obesity. SUA was negatively correlated with Bifidobacterium and positively correlated with VFA. SUA may be a mediating factor between decreased Bifidobacterium and increased visceral adipose tissue. Supplementation with Bifidobacterium might be a potential approach to reduce visceral adipose tissue.

## Supplementary Information

Supplementary Table 1.

## Data Availability

The datasets used and/or analyzed during the current study available from the corresponding author on reasonable request.
