# Supplementary material for: The abundance of bifidobacterium in relation to visceral obesity and serum uric acid
Source: Sci Rep. 2022 Jul 29;12:13073. doi: 10.1038/s41598-022-17417-3 (PMC9338261; doi:10.1038/s41598-022-17417-3)
Supplement: Supplementary file 1 — Supplementary Table 1. [file 41598_2022_17417_MOESM1_ESM.docx]

**Supplementary table 1** Primers used in this study

| Target group | Sequence (5'→3') | Annealing temperature(°C) |
| --- | --- | --- |
| *Lactobacillus* | F：GAGGCAGCAGTAGGGAATCTTC | 60.48 |
|  | R:GGCCAGTTACTACCTCTATCCTTCTTC | 62.09 |
| *Bifidobacterium* | F：CTCCTGGAAACGGGTGG | 56.76 |
|  | R:GGTGTTCTTCCCGATATCTACA | 57.07 |
| *F. prausnitzii* | F：GGAGGAAGAAGGTCTTCGG | 57.89 |
|  | R:AATTCCGCCTACCTCTGCACT | 61.52 |
| *C. butyricum* | F：ATGCAAGTCGAGCGAKG | 60.00 |
|  | R:TATGCGGTATTAATCTYCCTTT | 60.00 |
| *C. leptum* | F：GCACAAGCAGTGGAGT | 53.81 |
|  | R:CTTCCTCCGTTTTGTCAA | 52.40 |
| *E. rectale* | F：CGGTACCTGACTAAGAAGC | 54.75 |
|  | R:AGTTTCATTCTTGCGAACG | 54.38 |
| *Bacteroides* | F：GAGAGGAAGGTCCCCCAC | 66.67 |
|  | R:CGCTACTTGGCTGGTTCAG | 57.89 |
| *Enterococcus* | F：CGTGCGAACATGACCGATAT | 58.51 |
|  | R:CGAAACGGCCATTAACCAAC | 58.03 |
| Enterobacteriaceae | F：GGTAGAGCACTGTTTTGGCA | 58.40 |
|  | R:TGTCTCCCGTGATAACTTTCTC | 57.55 |
| *Atopobium* | F：GGGTTGAGAGACCGACC | 56.11 |
|  | R:CGGRGCTTCTTCTGCAGG | 57.80 |
